# Current Perspectives on Synthetic Compartments for Biomedical Applications

**DOI:** 10.3390/ijms23105718

**Published:** 2022-05-20

**Authors:** Lukas Heuberger, Maria Korpidou, Olivia M. Eggenberger, Myrto Kyropoulou, Cornelia G. Palivan

**Affiliations:** 1 Department of Chemistry, University of Basel, Mattenstrasse 24a, BPR 1096, 4058 Basel, Switzerland; lukas.heuberger@unibas.ch (L.H.); maria.korpidou@unibas.ch (M.K.); olivia.eggenberger@unibas.ch (O.M.E.); myrto.kyropoulou@unibas.ch (M.K.); 2 NCCR-Molecular Systems Engineering, Mattenstrasse 24a, BPR 1095, 4058 Basel, Switzerland

**Keywords:** polymers, compartments, biomedical

## Abstract

Nano- and micrometer-sized compartments composed of synthetic polymers are designed to mimic spatial and temporal divisions found in nature. Self-assembly of polymers into compartments such as polymersomes, giant unilamellar vesicles (GUVs), layer-by-layer (LbL) capsules, capsosomes, or polyion complex vesicles (PICsomes) allows for the separation of defined environments from the exterior. These compartments can be further engineered through the incorporation of (bio)molecules within the lumen or into the membrane, while the membrane can be decorated with functional moieties to produce catalytic compartments with defined structures and functions. Nanometer-sized compartments are used for imaging, theranostic, and therapeutic applications as a more mechanically stable alternative to liposomes, and through the encapsulation of catalytic molecules, i.e., enzymes, catalytic compartments can localize and act in vivo. On the micrometer scale, such biohybrid systems are used to encapsulate model proteins and form multicompartmentalized structures through the combination of multiple compartments, reaching closer to the creation of artificial organelles and cells. Significant progress in therapeutic applications and modeling strategies has been achieved through both the creation of polymers with tailored properties and functionalizations and novel techniques for their assembly.

## 1. Introduction

Mimicking the structure and function of materials found in nature is a well-known strategy for developing materials with structures and functions of interest. Vesicles are nature’s simplest compartments (e.g., organelles inside cells) and have existed since the first cells. While cell membranes are based on phospholipids, as they are natural amphiphiles, vesicle-forming molecules can also be synthetic. Copolymers are alternative amphiphiles that self-assemble into macromolecular assemblies [1,2]. Block copolymers, containing hydrophilic and hydrophobic regions that can be arranged in different repeating orders, have properties that can be controlled through chemical modification to support a desired application [3]. Based on the number of blocks used, amphiphilic polymers are referred to as di- or triblock copolymers, AB or ABA, respectively, with A representing the hydrophilic and B the hydrophobic block. Polyelectrolytes, polymers containing ionic or ionizable groups, also have the ability to self-assemble into synthetic compartments such as layer-by-layer (LbL) capsules or PICsomes [4]. Various molecular properties of the polymer blocks such as polydispersity, charge, block ratio and length, and molecular weight are essential to induce the supramolecular assemblies formed thereof. The addition of biomolecules changes the properties of the polymer membranes, the integration of phospholipids can change membrane mechanical properties [5,6,7], and membrane proteins can alter membrane permeability [8] and even provide the desired functionality via their intrinsic bioactivity [9,10].

In this review, we give an overview of polymer compartments and indicate how they have been engineered when medical applications are the aim, ranging from simple drug delivery systems to artificial organelles and cells. After starting with the basic concepts of self-assembly and methods for the formation of polymer compartments, we give details on the complex requirements for medical applications and eventually cover both novel examples serving as models and systems already designed for specific medical applications. With a focus on polymer vesicles (polymersomes (<1 μm in diameter) and giant unilamellar vesicles (GUVs, 1–100 µm in diameter)), we also present other polymer compartments such as hybrid vesicles, LbL capsules, capsosomes, and PICsomes. While drug delivery systems based on polymer compartments constitute a fast-growing field, we only briefly present on these, as excellent reviews on this topic can be accessed elsewhere [11,12,13,14]. Eventually, we highlight open questions in the field and discuss how they might be solved by future studies.

## 2. Generation of Synthetic Compartments

A hollow cavity surrounded by a synthetic barrier (membrane, layers of polymers) represents the common architecture that forms single compartments. When more complex structures are the aim, there are two different approaches: (i) encapsulation of small nanocompartments in GUVs to generate compartments-in-compartments and (ii) zipping together compartments to generate clusters or networks [15,16,17,18].

Self-assembly of amphiphilic block copolymers in aqueous solutions can result in several types of nano- or micrometer-sized structures such as micelles, tubes, or vesicles (polymersomes and GUVs) [19,20]. Self-assembly is driven by noncovalent interactions such as the hydrophobic effect or electrostatic interactions [21]. A list of polymers commonly utilized to form compartments with a hollow-sphere architecture is presented in Table 1.

**Scheme 1:**
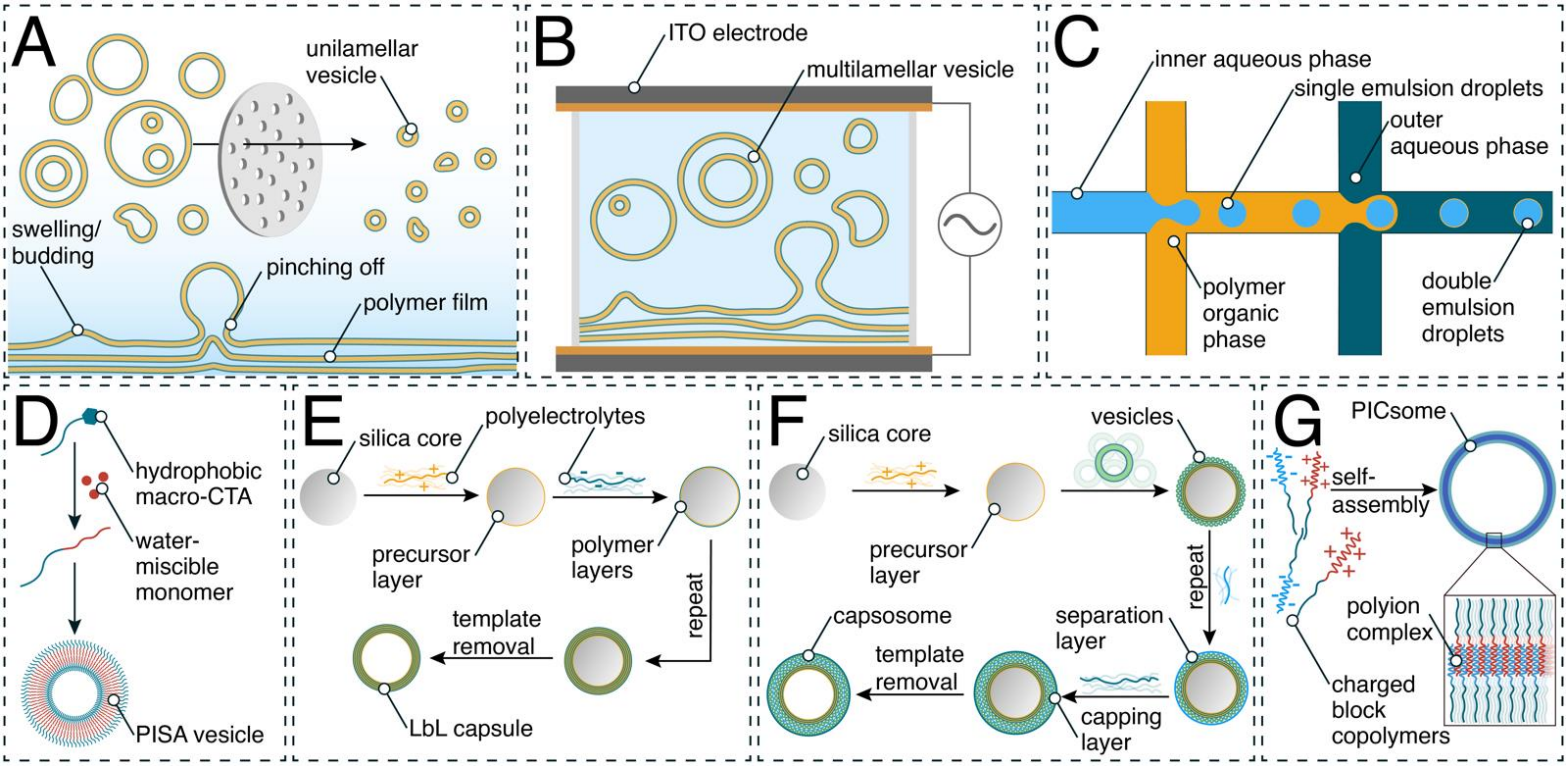
Schematic representation of selected production methods of synthetic compartments: (**A**) film rehydration and subsequent extrusion of block copolymers; (**B**) electroformation; (**C**) double emulsion formation with a microfluidic setup; (**D**) polymerization-induced self-assembly (PISA); (**E**) layer-by-layer (LbL) assembly; (**F**) assembly of capsosomes; and (**G**) PICsomes.

Here we present the most established self-assembly methods for nano- and micro-sized polymer vesicles (Scheme 1) and briefly describe how layer-by-layer assembly is used to generate capsules and capsosomes. In methods such as solvent switch, the block copolymer is dissolved in a water-miscible organic solvent. This is followed by the dropwise addition of an aqueous buffer to slowly replace the organic phase [22,23]. Contrarily, the cosolvent method is based on the dropwise addition of a copolymer solution to an aqueous buffer phase, which induces the self-assembly process of copolymers [24]. One drawback of these methods is the residual presence of organic solvent in the final solution, which is undesirable for biologically relevant applications. The film rehydration method (Scheme 1A) takes place in a more biocompatible manner, as the organic solution of the copolymer is completely dried, forming a film. The thin copolymer film is subsequently rehydrated with an aqueous solution, inducing the self-assembly process and resulting in supramolecular architectures (micelles, polymersomes, worm-like assemblies). When the hydrophilic–hydrophobic ratio *f* of the copolymers is in the range of 35 ± 10% [25], vesicles are the favored supramolecular assembly, even if the copolymers have a rather high polydispersity index (PDI) [17,26]. Film rehydration is well suited for loading polymersomes with sensitive molecules, such as enzymes and proteins, during the self-assembly process [27]. However, as this process is based on statistic loading of the desired molecules present in the rehydration solution, the encapsulation efficiency is highly dependent on their solubility and usually ranges between 5 and 20% for encapsulation of a single type of high-molecular-weight molecule, such as proteins and enzymes, and decreases even further for coencapsulation of two types of proteins [28,29,30].

When the aim is giant unilamellar vesicles, methods used for polymersome generation are joined by other methods, including electroformation. The electroformation technique (Scheme 1B) is based on the spontaneous swelling of a dried block copolymer film that has been deposited on two electrodes of indium tin oxide (ITO)-coated glass or platinum and the consequent formation of GUVs in the presence of an aqueous solution stimulated by an electric field [17,31]. This method has a high yield of assemblies with high levels of unilamellarity [32] but is limited by a broad size dispersity and can be used only for uncharged amphiphiles to avoid electrostatic interactions affecting the self-assembly process [33]. Emulsion centrifugation is a method for the formation of GUVs wherein a water-in-oil emulsion suspension is transferred into a water phase by centrifugation [34]. The single emulsions cross a polymer monolayer at the water/oil interface and are coated by a second monolayer, resulting in a bilayered GUV. Microfluidic technology represents a step forward that has been recently used for high-throughput GUV formation with a narrow size distribution based on microdevice channel sizes and junction design [34,35,36]. Polymer-stabilized water–oil–water (w/o/w) double emulsions are used to form GUVs. An aqueous solution is enclosed in a layer of organic phase, consisting of the amphiphilic block copolymer dissolved in a volatile organic solvent (Scheme 1C). A flow of outer aqueous solution then pinches off the double emulsion droplets. Double emulsions can be created with a multitude of different microfluidic designs, such as glass capillaries [36,37] or molded microchannels in varying layouts [38,39,40]. Subsequent evaporation of the volatile organic solvent leads to the formation of a GUV with a polymer membrane. An extremely high encapsulation efficiency (99%) can be obtained by including in the inner flow the molecules (enzymes, proteins) desired to be encapsulated within the generated GUVs. The molecules planned to be entrapped in the membrane (biopores, membrane proteins) are also included in the aqueous flow at this stage [39]. Despite the high encapsulation efficiency and monodispersity, there are still limitations to using this method for GUV formation, such as its complexity and the necessity of specialized equipment.

An entirely different approach is that of polymerization-induced self-assembly (PISA), a technique that directly produces nanoassemblies during the block copolymer synthesis (Scheme 1D) [41,42,43]. PISA’s principle is based on the chain extension of a soluble precursor polymer block in a suitable solvent with the simultaneous use of a second polymerizing monomer, resulting in the formation of an insoluble second block, yielding polymersomes [41,42,43]. The advantage of the method is its efficiency, as it combines synthesis with self-assembly and is characterized by monodispersity. On the other hand, this method is limited by decreased colloidal stability in the presence of ionic surfactants. Furthermore, PISA can only be applied for a small selection of monomers [44].

Polymer capsules are frequently formed through LbL deposition (Scheme 1E), a technique that involves the controlled adsorption of polymer layers on a sacrificial colloidal particle, alternating between oppositely charged materials, i.e., one layer of negatively charged polymer is followed by one of positively charged polymer [45,46,47]. Once the appropriate number of layers has been applied, the system is submerged in a solution designed to either dissolve the template or simply detach the vesicle membrane, allowing one to separate out the freshly formed vesicles. LbL capsule formation is simple yet versatile in terms of compartment size and components, including their functionality possibilities [48,49]. However, this method is limited by its dependence on the sacrificial template. Formed through a similar technique, capsosomes are capsules of which the membranes are composed of smaller vesicles. Their fabrication involves the deposition of an initial number of polymer layers onto a colloidal particle of specified size. Next, one or multiple layers of liposomes are attached, segregated by a polymeric separation layer, followed by a final deposition of several protective polymer layers (Scheme 1F) [50,51]. Assemblies of a different type, PICsomes, are the vesicular form of polyion complex (PIC) particles and are self-assembled through electrostatic interactions [23,26]. PICsomes are composed of oppositely charged block copolymers (Scheme 1G), and their membranes are semipermeable, especially to hydrophilic solutes. A major advantage of PICsomes is their facile formation—they naturally self-assemble through the mixing of their charged components [52]. One disadvantage of PICsomes is that their components typically need to be combined at equal concentrations to result in a charge-neutral vesicle, though recent experimentation has shown that charge-balance-sensitive materials can be formed by varying this ratio [53].

**Table 1:** Polymers used for biomedical applications.

Polymer	Method of Self-Assembly	Characteristics
Carbohydrate-*b*-PPG	Direct hydration method [54]	Forms capsosomes, inherently permeable to low-molecular-weight compounds
Chitosan	Sonication-assisted mixing (capsules) [55], LbL [56]	Biocompatible, natural polymer
CTAB	LbL [56]	Surfactant, forms micelles in the absence of another polymer
PA/DEX	LbL [57]	Biocompatible polysaccharide (anionic)
P(OEGMA300-*grad*-HPMA)	PISA [43]	Biocompatible assembly, monomers and a macromolecular precursor need to be: (i) solvophilic and (ii) compatible with each other
PA/PLA	LbL [57]	Biocompatible cationic polyelectrolyte
PAA	LbL [58]	Anionic polyelectrolyte
PAH	LbL [59,60]	Cationic polyelectrolyte
PAMAM	Mixing (PICsomes) [61]	Dendrimer (branched structure)
P(Asp-AP)	Mixing (PICsomes) [62,63,64]	Anionic polyelectrolyte, forms PICsomes, cannot form vesicles on its own
PATK	Mixing (PICsomes) [65]	Cationic polyelectrolyte
PBd-*b*-PEG	Double emulsion microfluidics [37]	Biocompatible
PBd–*b*-PEO	Emulsion centrifugation [34], Electroformation [66], Film rehydration [67]	Pure or as hybrid (with POPC) polymersomes for membrane protein insertion, assembly of asymmetric polymer/lipid (POPC) hybrid membranes
PBO-*b*-PG	Microfluidic double emulsion, solvent switch [68]	Biocompatible
PCL-*b*-P[Glu-stat-(Glu-ADA)]	Solvent switch [69]	Biodegradable, bone-targeting
PCL-*b*-PTrp-*b*-P(Lys-statPhe)	Solvent switch [70]	Biocompatible, biodegradable, antibacterial
PDMS-*b*-heparin	Film rehydration [71]	Forms polymersomes in combination with PMOXA-*b*-PDMS-*b*-PMOXA, forms micelles by itself
PDMS-*g*-PEO	Electroformation, Film rehydration [72]	Pure or as hybrid (with PC) polymersomes and GUVs for membrane protein insertion
PEG-*b*-PCL	Electroformation [73], film rehydration [74]	Multidomain membrane formation with lipids (DPPC)
PEG-P(CLgTMC)	Direct hydration method [75]	Biodegradable, intrinsic fluorescence
PEG-*b*-P(CPTKMA-*co*-PEMA)	Solvent exchange method [76]	Biocompatible, conjugated with campthothecin
PEG-GPLGVRG-PCL-PGPMA	Film hydration method [77]	Biocompatible, MMP-cleavable peptide and CPP-mimicking polymer
PEG-*b*-PHPMA	PISA [78]	Highly hydrated membrane, size-selective transport of molecules
PEG-*b*-PIC	Solvent exchange [79]	Biocompatible, iodine-rich for SPECT/CT and radioisotope therapy
PEG-*b*-PLA	Film rehydration [74], double emulsion microfluidics [37]	Forms polymersomes with and without lipid mixing, biodegradable
PEG-*b*-polypeptide	Mixing (PICsomes) [80]	pH-responsive, biocompatible
PEG-*b*-PAsp	Mixing (PICsomes) [62,64]	Linear polymer, forms PICsomes, micelles or hydrogels, biocompatible
PEG-*b*-PS	Solvent switch method [81,82]	Biocompatible, formation of stomatocytes, rigid assemblies
PEI-*b*-PDLLA	Microfluidic double emulsion [83]	Biocompatible, cationic assemblies, can form polymer stomatocytes
PEO-*b*-PBO	Film rehydration [84]	Forms asymmetric polymersomes
PEO-*b*-PCL	Emulsification-induced assembly [85]	Low interfacial tension solvent or SDS is needed to control the assembly
PEO-*b*-PCL-*b*-PMOXA	Film rehydration [86]	Rehydration at 62 °C due to the semi crystalline nature of the PCL block
PEO-*b*-P(CMA-stat-DEA-stat-GEMA)	Solvent exchange method [87]	Biocompatible, CMA photocrosslinking stabilization
PEO-*b*-PEHOx-*b*-PEtOz	Solvent switch, film rehydration [26]	Asymmetric membrane, can be used for directed protein insertion
PEO-*b*-PPO-*b*-PEO (Pluronics L121)	Double emulsion microfluidics [37]	Assembly via DNA linkage
PiB-*b*-PEG	Freeze–thaw extrusion [88]	Biocompatible, high chemical and thermal stability
PLys	Mixing (PICsomes) [89]	Cationic polyelectrolyte
PMA	LbL [60]	Labor-intensive LbL assembly
PMOXA-*b*-PDMS	Film rehydration [90,91], microfluidic double emulsion [39]	Formation of nano- and micro-sized vesicles in biocompatible, aqueous conditions, various channels and proteins can be inserted
PMOXA-*b*-PDMS-*b*-PMOXA	Film rehydration [71,92]	Formation of nano and micro-sized vesicles in biocompatible, aqueous conditions, various channels and proteins can be inserted
PMPC-*b*-PDPA	Film rehydration [84,93]	Formation of (asymmetric) polymersomes, can be electroporated
POEGMA-*b*-P(ST-co-VBA)	PISA [41]	Biocompatible assembly, monomers and a macromolecular precursor need to be: (i) solvophilic and (ii) compatible with each other
Poly(dopamine)	LbL [94]	Simplified LbL capsule formation
PS-*b*-PEO	Emulsification [95]	High capacity of ammonia capture in bile salt-containing buffer
PSMA-PBzMA	PISA [42]	Biocompatible assembly, monomers and a macromolecular precursor need to be: (i) solvophilic and (ii) compatible with each other
PSS-*b*-PEO-*b*-PSS	Mixing (PICsomes) [61]	Forms PICsomes with loops within the membrane when combined with poly(amidoamine) dendrimers
PVP	LbL [96]	Work-intensive LbL assembly

### 2.1. Surface Functionalization of Polymer Compartments

The external surfaces of compartments can be functionalized to attach different molecules with the aims of increasing their stability by cross-linking [97], attaching them on solid support to generate surfaces with nano/micro texture [98], or targeting specific biolocations (e.g., tumors [99,100] or specific organs [59,69,101,102]).

One approach to achieve external attachment of molecules is to introduce the desired chemical or reactive groups to the end of the hydrophilic copolymer block during the polymerization process [103]. The functional end groups often include hydroxyl [91,103], amine [104], and N-hydroxysuccimidyl esters [105]. Then, by using a mixture of the end-functionalized block copolymer with a nonfunctionalized copolymer at the desired molar ratio and the methods described above, compartments bearing functional groups are formed. The molar ratio in which the functionalized copolymer is mixed is optimized to be both high enough to support the desired fraction of attached molecules after polymersomes/GUVs formation and low enough to not impede their formation. Using only functionalized polymers often leads to the formation of undesired micelles or aggregation phenomena.

**Figure 1:**
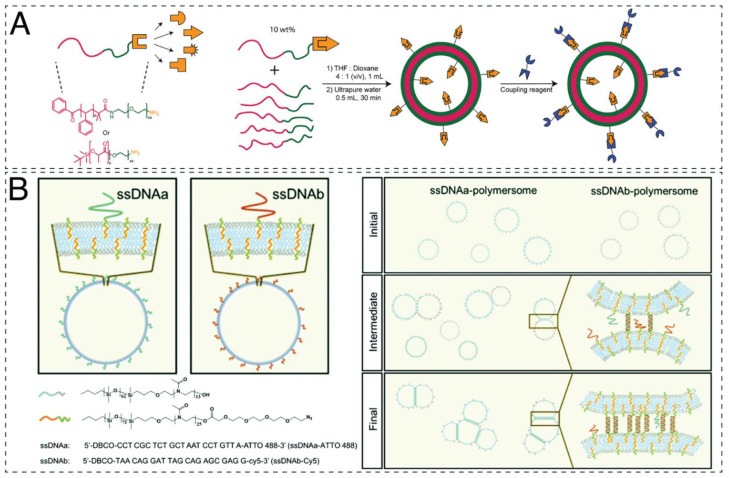
(**A**) Structure of the modular PEG-*b*-PS and PEG-*b*-PDLLA polymer with amine moiety used for attaching various functionalities. Functionalized PEG-*b*-PS and PEG-*b*-PDLLA polymers are self-assembled into polymersomes upon the addition of water. Functionalized polymers can be used for further decoration of polymersomes. Adapted with permission from [117]. Copyright 2020 American Chemical Society. (**B**) Schematic representation of DNA-decorated polymersomes; chemical structures of the block copolymers and ssDNA sequences used for assembly and step-by-step clustering process of ssDNA-polymersomes. Polymersomes link via DNA hybridization, which is followed by migration of the nonhybridized ssDNA and the formation of a DNA bridge area. Adapted with permission from [120]. Copyright 2020 Royal Society of Chemistry.

Once the compartments expose the functional groups at their external interface to the environment, they can be used to further attach the desired molecules either by covalent attachment or by using molecular recognition. Covalent attachment of molecules at the compartment surface involves different chemical approaches. For example, strategies based on Cu^2+^-free click chemistry are achieved by: (i) azide–alkyne cycloaddition [106,107,108,109,110], (ii) maleimide and thiol-ene [111,112,113,114,115], and (iii) amine coupling [114,116,117,118]. Using a combination of strain-promoted azide–alkyne cycloaddition (SPAAC) and thiol-ene reactions, the surface modification of poly(2-methyl-2-oxazoline)-*block*-poly(dimethylsiloxane) (PMOXA-*b*-PDMS) polymersomes served to immobilize polymersomes on surfaces [111,112,119]. The functionalization of PMOXA-*b*-PDMS polymersomes with 4-formylbenzoate facilitated the attachment of hydrazone(HyNic)-functionalized antibodies for biotin [116]. Moreover, a recent study reported a general methodology for the surface functionalization of polymersomes. Starting from poly(ethylene glycol)-*block*-poly(styrene) (PEG-*b*-PS) and poly(ethylene glycol)-*block*-poly(D,L-lactic acid) (PEG-*b*-PDLLA) polymers and activating the amine functionalization, the authors were able to form surface-functionalized polymersomes featuring various functional groups (Figure 1A) [117].

While it is more common to functionalize the surface of polymersomes than those of PICsomes and LbL capsules, some recent examples exist where the PICsome surface or the last layer of a capsule’s membrane was functionalized or designed to be functionalizable. For example, a common way to add antifouling properties to a capsule is the addition of PEG to the final polymer layer. This allows the capsules to circulate within the bloodstream for the length of time necessary for them to reach their intended target [121]. PICsomes were functionalized with cyclic arginine-glycine-aspartic (cRGD) peptides via covalent attachment of the N-terminal cysteines of the cRGD peptides to aldehyde groups on the PICsome surface in order to specifically recognize integrins expressed at high levels in the neovascular system [122].

A second option for attaching the desired molecules at the surface of compartments is to use molecular recognition as the driving force. Among the most effective pairs of molecules involved in molecular recognition are biotin–streptavidin proteins [118,123,124] and nucleic acid hybridization [118]. A highly specific conjugation method was mediated by oligonucleotide sequences such as single-stranded DNA (ssDNA) [37,118]. In detail, azide-exposing PMOXA-*b*-PDMS polymersomes enabled coupling of dibenzocyclooctyne (DBCO)-derivatized ssDNA or its complementary oligonucleotide. Because of the specificity of complementary base pairing, DNA can be used as a means to direct the self-organization of polymersomes into more complex structures (Figure 1B) [125].

The combination of increased stability and enhanced flexibility of polymersomes with the precision of DNA hybridization leads to the formation of clusters that are both biocompatible and stable in physiological conditions. In particular, when DNA-zipped polymersome clusters were incubated with DNase I, a well-known endonuclease, the clusters maintained their structure. Additionally, polymersome–DNA clusters remained stable for up to 10 h in cell medium [120].

### 2.2. Assemblies of Compartments

Compartments have been used to generate more complex assemblies, either by encapsulating nanoassemblies into GUVs (compartments-in-compartments architecture) [67,71,92] or by the association of compartments and formation of networks and clusters [91,103,120]. Compartment-in-compartment architectures include various combinations of functional polymer or lipid membranes and multilayer capsules enclosed in a GUV membrane within which they can interact. The encapsulation of multiple compartments, each with distinct membranes and functionality, into a GUV membrane forms a system of increased complexity that mimics the eukaryotic cell. Such systems allow for the study of cascade reactions or signaling pathways between the environment of the GUVs and their inner compartments or in between them. While such systems allow more controlled cascade reactions, the encapsulation efficiency of nanocompartments into GUVs is still low, which generates issues of sensitivity [126]. On the contrary, clusters, which are formed by zipping together compartments into a structure similar to that of cell colonies, have an architecture that supports an increase in the encapsulation efficiency while allowing for the location of different molecules in segregated spaces at the nano scale [91,103,120]. Bioconjugation based on nucleic acid hybridization has been applied to both liposomes [127] and polymersomes [125]. For example, polymersomes decorated with exposed ssDNA were zipped to polymersomes exposing complementary ssDNA strands to form clusters that remained stable in vitro and in vivo. Another example illustrated that by using DNA as a polymer block, polymethyl acrylate-*block*-DNA (PMA-*b*-DNA) and poly(butadiene)-*block*-poly(ethylene oxide)-*block*-DNA (PBd-*b*-PEO-*b*-DNA) supported the formation of DNA-bearing giant vesicles (GUVs) that were interconnected via DNA interactions and assembled into a “vesicular island” structure [128]. A critical aspect of polymersome clusters is the control of their size to avoid aggregation. This control is achieved by optimizing the number of ssDNA strands exposed at the surface of polymersomes to support the zipping process while keeping the number of unbound ssDNA strands low [129]. The unique advantages of clusters of compartments are the possibility of having segregated spaces for different molecules and an increase in the encapsulation efficiency compared with that of compartments-in-compartments. Both types of complex compartments support multifunctionality as an essential step for advanced applications.

## 3. Requirements for Compartments to Be Used in Biomedical Applications

For any biologically relevant material, the transition from the research laboratory to a clinical setting is a complex procedure. This is particularly true when the final application is in the field of medicine, pharmaceutical production, or personal care [130,131,132]. In the case of compartments, there is a complex set of requirements, first for the copolymers used to generate the compartments and then for the compartments themselves. Therefore, for an amphiphilic block copolymer or a polyelectrolyte to be used in biomedical applications some crucial aspects need to be considered: (i) the polymers should be able to self-assemble in aqueous solutions [19,23,86]; (ii) they need to be nontoxic and biocompatible [133]; (iii) they need to have the appropriate physical characteristics (i.e., flexibility, membrane thickness) [134] in order to facilitate the functional insertion, encapsulation, and reconstitution of biomolecules [135,136]; (iv) control over their surface charge is crucial for the attachment of biomolecules or further in vivo applications [137]; (v) they need to maintain stability under physiological conditions [138,139] (i.e., in high temperature or salt concentration, their functionality must remain, and the availability of the encapsulated biomolecules must be sustained); and (vi) of particular import for therapeutic compartments is their ability to undergo endosomal escape so the therapeutic agent is not hindered by cells’ defense mechanisms and can arrive at the pathogenic site [140,141,142]. Besides these, one of the most limiting factors is the fate of copolymers in the body. Therefore, their biodegradability still remains a real challenge for various copolymers that fulfill the complex list of factors aforementioned.

Second, the compartments planned to be used in medical applications should fulfill their own set of requirements. While polymersomes, capsules, and capsosomes should preserve their integrity and act as catalytic compartments, they must also allow the molecular flow of substrates and products. Their membrane can be rendered permeable by biopores such as ion channels [135,143], peptide pores [30,144], DNA nanopores [93], and natural membrane proteins [139]. While many examples of functional protein reconstitution in polymeric membranes have been shown, the increased hydrophobic mismatch of polymeric membranes as compared with lipidic ones limits the choice of membrane protein. High membrane fluidity is essential for protein incorporation, as it allows integrating proteins into membranes that are several times thicker than the protein itself and helps the protein to overcome the size mismatch between its own hydrophobic region and the hydrophobic region of the polymer membrane [134]. Furthermore, the surface charge plays a crucial role in the in vivo functionality, biodistribution, and cellular uptake of the systems [145,146]. Specifically, positively charged compartments have been found to be better uptaken by cells and exhibit improved biodistribution [147,148,149], while negatively charged ones are usually less toxic and better at specific tissue targeting [149,150,151].

The surface functionalization of polymersomes, capsules, and capsosomes opens up many possibilities for the assembly of complex soft compartments. However, the attachment of functional groups changes the overall charge and might induce interactions with the environment or trigger aggregation, increasing the intrinsic toxicity. Additionally, the formation of clusters of compartments increases the size of the final assembly. This limits the possible applications mainly to the intercellular matrix [152,153]. Overall, use in medical applications requires both the polymer properties (e.g., toxicity, biocompatibility) and those of the compartment (e.g., membrane composition, functionalization, size, charge) to be carefully tailored.

## 4. Applications of Compartments in the Biomedical Field

Numerous studies have investigated the benefits and limitations of nanometer-sized compartments for their use in imaging, therapeutics, and theranostics. As a first step, in vitro studies have explored the activity and cytotoxicity of the systems for these applications, while in vivo studies have highlighted their suitability and investigate in-depth parameters for clinical translation. Meanwhile, micrometer-sized vesicles have facilitated the modeling of cells, a crucial step in bottom-up synthetic biology aiming to bring further insights to real-life processes. An overview of biomolecules that have been used within compartments or in their membranes is presented in Table 2.

### 4.1. Imaging and Theranostic Applications

Fluorescence and bioluminescence are powerful optical techniques that are often used in medical imaging. However, limitations associated with low specificity and solubility or high toxicity of imaging agents have led to an increased interest in designing nanocompartments for such applications. While nanoparticles are promising candidates for theranostic agents, they are not discussed here, as thorough reviews have been published recently [164,165,166].

**Figure 2:**
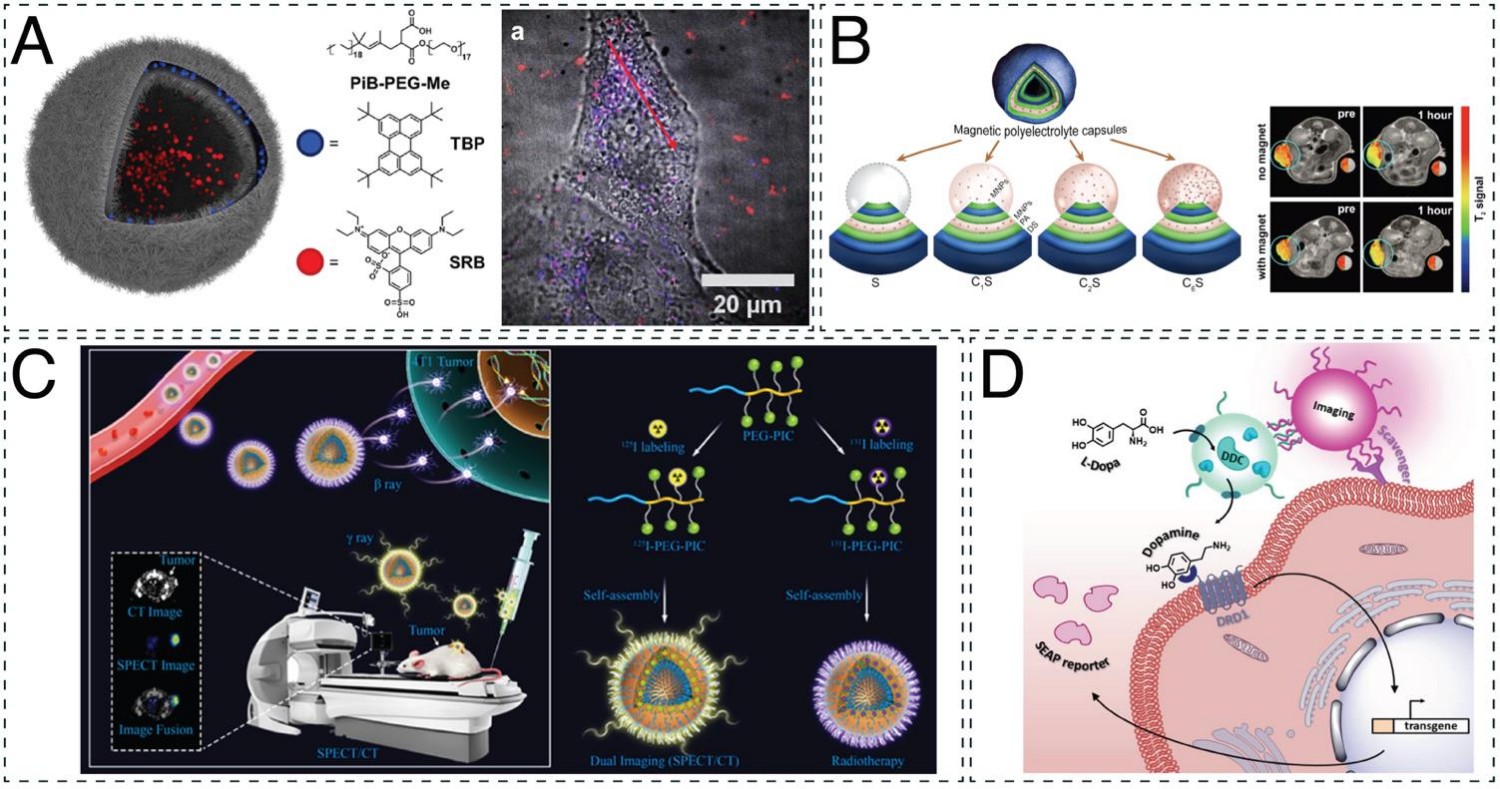
Imaging and theranostic applications of nanocompartments. (**A**) Dual fluorescence of polymersomes for optical imaging. Adapted with permission from [168]. Copyright 2018 Elsevier. (**B**) Magnetic LbL capsules for cancer targeting and treatment by magnetic resonance imaging (MRI). Adapted with permission from [57]. Copyright 2021 Ivyspring International Publisher. (**C**) Radiolabeled iodine-rich polymersomes for single-photon emission computed tomography (SPECT)/computed tomography (CT) dual imaging and cancer therapy. Reprinted with permission from [170]. Copyright 2019 American Chemical Society. (**D**) Theranostic DNA-zipped polymersome clusters composed of two nanocompartments: a therapeutic, dopa decarboxylase (DDC)-containing polymersome and an imaging, DY-633-containing polymersome. Reprinted with permission from [103]. Copyright 2020 John Wiley and Sons.

By forming polymersomes from polyisobutylene-monomethyl-*block*-polyethylene glycol (PiB-*b*-PEG) block copolymers, triplet–triplet annihilation-based molecular photon upconversion (TTA-UC) chromophores, palladium(II) tetraphenyl tetrabenzoporphyrin and 2,5,8,11-tetra(tert-butyl)perylene were entrapped and protected by natural antioxidants [167]. This system allowed red-to-blue upconversion in aerobic conditions, which is otherwise hindered. When these polymersomes were tested on lung carcinoma cells treated with a mixture of antioxidants in 1% oxygen, mimicking the low oxygen concentrations encountered in tumors, the upconversion emission was one order of magnitude higher, proposing a solution to the oxygen sensitivity of TTA-UC systems. Going a step further, PiB-*b*-PEG polymersomes containing one fluorescent probe in the polymer membrane and a second in the aqueous cavity served for dual fluorescent imaging (Figure 2A) [168]. In lung cancer cells, these polymersomes remained intact for 90 h postinjection and were not exocytosed but did reduce the cell proliferation. When injected in zebrafish embryos, they remained intact and active 96 h postinjection, were not excreted or degraded, and did not cause any animal death. In the same respect, the successful encapsulation of quantum dots in PMOXA-*b*-PDMS polymersomes shielded their toxic effects, as no cytotoxicity was observed when uptaken by HepG2 liver cancer cells [169]. Furthermore, they were found to be more stable than the respective liposome nanostructures which released the quantum dots in the cytoplasm of the cells. However, in comparison with free quantum dots, which are internalized rapidly but nonspecifically by living cells, the internalization of these polymersomes occurred over almost 17 h, which should be taken into consideration during the clinical translation of this system. To capitalize on the advantages of luminescence, polymersomes made of PMOXA-*b*-PDMS with encapsulated luciferase served for the in vitro production of strong and long-lasting luminescence, giving the system the potential to be used in biomedical imaging applications [160].

Moreover, a theranostic system can be established, offering the advantage of simultaneous diagnosis and treatment [114]. For example, poly(ether imide)-*block*-poly(D,L-lactide) (PEI-*b*-PDLLA) polymersomes were recently used for neuronal restoration treatment trackable by magnetic resonance imaging (MRI) [171]. Superparamagnetic iron oxide nanoparticles (SPIONs) and the siRNA targeting the Nogo-66 receptor (NgR) gene were encapsulated in these nanocompartments. Although the application of these polymersomes led to around five times less NgR protein expression, a 30% increase in neuronal differentiation of stem cells was shown in vitro. In an acute ischemic stroke rat model, the polymersomes promoted the recovery of the animals better than the control group (resulting in a lower infarct volume and modified neurological severity scores (mNSS)). Employing the same principle, LbL capsules composed of magnetite nanoparticles and layers of poly-L-arginine and dextran sulfate were used in cancer treatment (Figure 2B) [57]. These capsules were traceable and directed using an external magnetic field during MRI in a mouse breast cancer model. Although they exhibited a high spleen accumulation postinjection, when a magnetic field was applied, their accumulation in the tumor increased threefold. For single-photon emission computed tomography (SPECT)/computed tomography (CT) dual imaging, polymersomes based on radiolabeled poly(ethylene glycol)-*block*-poly(iodinated carbonate) (PEG-*b*-PIC) block copolymers were tested on immunodeficient mice for the theranostic treatment of 4T1 murine breast cancer (Figure 2C) [170]. The injection of these polymersomes was found to double the lifetime of the mice and reduce the tumor volume twofold when compared with the controls. However, it should be noted that after their intravenous injection, the polymersomes accumulated in healthy organs, with a 10% higher prevalence in the spleen than at the tumor site. For enhanced photodynamic therapy, polymersomes made from poly(ethylene glycol)-*block*-poly(caprolactone-*gradient*-trimethylene carbonate) amphiphilic block copolymers (PEG-*b*-P(CL*g*TMC)) and a terminal block of tetraphenylethylene pyridinium-modified trimethylene carbonate (PTMC) intrinsically fluorescent polymer were loaded with BODIPY photosensitizer [75]. These polymersomes showed enhanced mitochondrial targeting and tumor accumulation in A549 tumor-bearing nude mice, reducing the size of the tumor almost five times more than in control animals. However, these results were obtained after intratumoral injections of the polymersomes to the mice, which might be a limiting factor in clinical translation. Based on the same principle, polymersomes made from poly(ε-caprolactone)-*block*-poly(tryptophan)-*block*-poly(lysine-*stat*-phenylalanine) were able to exhibit intrinsic blue fluorescence for bacterial monitoring and antibacterial properties [70]. When tested on *Escherichia coli* and *Staphylococcus* *aureus*, the planktonic bacteria were killed within 4 h of polymersome administration, at a minimum effective concentration of 600 mg/mL for *E. coli* and 62.5 mg/mL for *S. aureus*. Clusters of PMOXA-*b*-PDMS polymersomes have also served as nanotheranostics agents (Figure 2D) [103]. Generated by the hybridization of DNA exposed on their surface and separately loaded with fluorescent dyes and enzymes, they successfully targeted human embryonic kidney cells. Interestingly, the ssDNA that remained free after the cluster formation facilitated their interaction with scavenger receptors on cells. Dopa decarboxylase (DDC), the enzyme that produces the neurotransmitter dopamine, was encapsulated separately from the fluorophore DY-633, resulting in clusters serving both imaging and therapeutic purposes (such as for the treatment of atherosclerosis).

### 4.2. Therapeutic Applications: From In Vitro to In Vivo

#### 4.2.1. In Vitro Studies

Before proceeding with any in vivo studies, in vitro evaluation of the toxicity and therapeutic potential of the nanocompartment system is of crucial importance. In one of the first studies of catalytic nanocompartments (CNCs) for antibacterial applications, penicillin acylase was encapsulated in PMOXA-*b*-PDMS-*b*-PMOXA polymersomes equipped with outer membrane protein F (OmpF) pores in the polymer membrane to facilitate the diffusion of the substrates and products [162]. These catalytic nanocompartments produced antibiotics locally and on demand and successfully inhibited the growth of *E. coli* cells. Antibacterial activity against *E. coli* and *Pseudomonas aeruginosa* was also exhibited by LbL capsules made of triclosan@cetyltrimethylammonium bromide micelles incorporated in dextran aldehyde polyelectrolyte multilayers [56]. These capsules inhibited bacterial growth in the short term (24 h) and the long term (up to 30 days), suggesting a means of extending the life of antimicrobial coatings. However, the importance of OmpF membrane permeabilization was further established in other PMOXA-*b*-PDMS or PMOXA-*b*-PDMS-*b*-PMOXA catalytic nanocompartments, designed for enzyme replacement therapy [16,159]. When urate oxidase (UOX) or HRP was loaded in polymersomes equipped with OmpF pores, the detoxification of uric acid and prevention of H_2_O_2_ accumulation took place in kidney-derived HEK293T cells as a first step towards the treatment of gout and oxidative stress [16]. Similarly, a cascade reaction in situ inside epithelial cells of adenocarcinoma and myoblasts served for the production of cyclic guanosine monophosphate (cGMP), a second messenger molecule involved in a number of pathologies [159]. Inducible nitric oxide synthase (iNOS) and soluble guanylyl cyclase (sGC) were encapsulated in separate nanocompartments, and the production of cGMP was monitored by measuring the cytoplasmic calcium levels. The highest response was recorded when both of the nanocompartments were present, highlighting their potential to influence cell physiology. However, it should be noted that OmpF allowed for the diffusion of molecules only up to 600 Da, limiting the possible applications of permeabilized polymersomes [90]. Apart from OmpF, melittin biopores can also be used for membrane permeabilization [161]. Melittin-permeabilized polymersomes made from PMOXA-*b*-PDMS encapsulating β-glucuronidase were prepared to invert the glucuronidation of drugs in situ. When tested in hepatocellular carcinoma cells, they were noncytotoxic, internalized, and successfully produced the drug hymecromone over 24 h when given its glucuronide conjugate. However, when the retention of the reaction products inside the cavity is crucial, no membrane permeabilization is necessary, and the catalyst and substrate can be coencapsulated. For example, PMOXA-*b*-PDMS polymersomes encapsulating tyrosinase and L-DOPA/dopamine, the precursors of melanin/polydopamine (PDA), were incubated for 24 h in order for their polymerization to occur. The resulting melanosome mimics had the ability to cross the cellular membrane, localize around the nucleus, and offer photoprotection to immortalized human keratinocytes (HaCaT cells) under UV irradiation [163].

**Figure 3:**
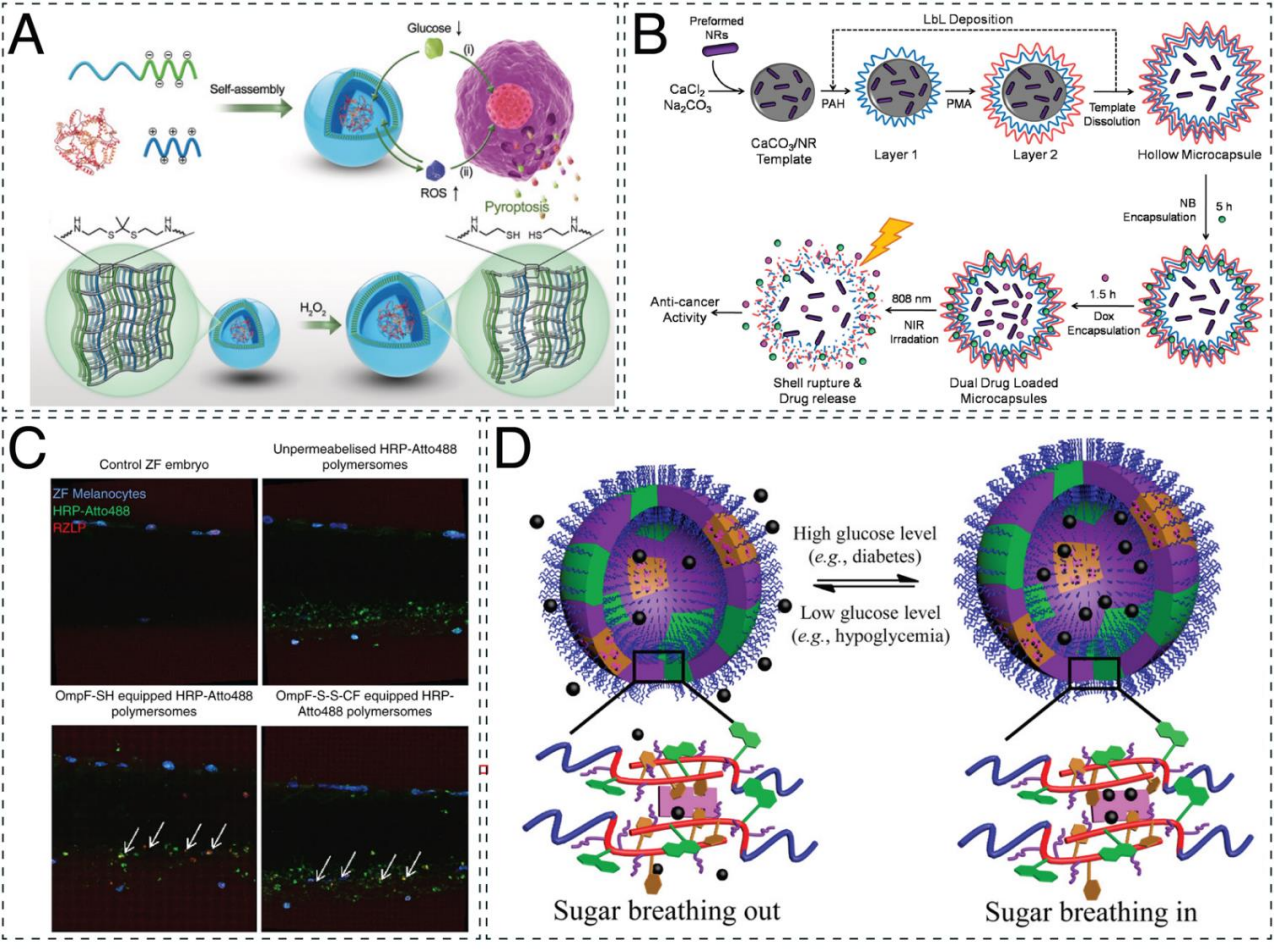
Therapeutic applications of nanocompartments. (**A**) Glucose oxidase -loaded PICsomes for inducing cell death by pyroptosis. Adapted with permission from [65]. Copyright 2020 John Wiley and Sons. (**B**) Layer-by-layer microcapsules for cancer chemophotothermal therapy. Reprinted with permission from [60]. Copyright 2020 Elsevier. (**C**) Biodistribution and activity of HRP-encapsulating polymersomes, acting as artificial organelles in a zebrafish embryo. Adapted with permission from [138]. Copyright 2018 Springer Nature. (**D**) Glycopolymersomes for regulating glucose levels for the treatment of diabetes mellitus. Adapted with permission from [87]. Copyright 2017 American Chemical Society.

Clusters of CNCs encapsulating glucose oxidase (Gox; Gox-CNCs) and lactoperoxidase (LPO; LPO-CNCs) can be used for a cascade reaction that functions as protection from lung infections, treatment of hyperglycemia, and reactive oxygen species (ROS) therapy [91]. The clusters were tested on lung carcinoma epithelial cells, where they successfully consumed glucose. When amyloglucosidase was attached to the outer membrane of the Gox-CNCs, the cell viability was no different from that of Gox-LPO-CNCs, and the cells were able to metabolize amylose. PICsomes have also been used for the encapsulation of Gox [65]. These nanocompartments were made of poly([2-[[1-[(2-aminoethyl)thio]-1-methylethyl]thio]ethyl]-α,β-aspartamide) (PATK) and PEG-*block*-poly(α,β-aspartic acid) (PEG-*b*-Pasp) and tested on two breast cancer cell lines and human fibrosarcoma cells for the treatment of cancer by pyroptosis (Figure 3A). The increased cell death from pyroptosis when cells were incubated with these nanocompartments was investigated and proven by extensively analyzing the cell morphology and detecting higher levels of DNA damage and release of intracellular components in treated than in untreated cells.

Apart from polymersomes and PICsomes, LbL capsules have been investigated for their in vitro efficiency in chemophotothermal therapy [60] and oxidative stress [59]. LbL capsules made from poly(allylamine hydrochloride) (PAH) and poly(methacrylic acid) (PMA) coencapsulated doxorubicin hydrochloride with gold nanorods, as well as nimbin within the layers of the membrane (Figure 3B) [60]. Upon near-infrared (NIR) irradiation, the increased temperature of the gold nanorods led to the formation of pores and ultimate rupture of the vesicles, causing almost 90% of the encapsulated material to be released within 30 min. In leukemia monocytes, the cell death was calculated at 99%, although 50% cell death was still observed without NIR irradiation. For reducing oxidative stress, LbL capsules with tannic acid (TA) surface functionalization were successfully tested in an in vitro inflammation model [59]. Layers composed of PAH, dextran sulfate, and TA had the ability to scavenge both H_2_O_2_ and radical -OH while having no negative effect on the nucleus pulposus cells that they treated. While the capsules did function to reduce the concentration of H_2_O_2_, they were less efficient than free enzymes because of the barrier between enzyme and substrate in the form of the polymer shell. Although LbL capsules have exhibited some very promising results when tested in vitro, it is important to bear in mind that their large size (2–5 μm) limits their in vivo use and further application.

Polymer stomatocytes are bowl-shaped polymersomes that have the ability to entrap catalytic cargo in their stomata [172,173]. When the cargo is appropriately selected (e.g., encapsulated Gox and catalase) and O_2_ is generated, the stomatocytes act as nanomotors and are able to perform slow self-propelled motion [174]. This feature has been investigated for its potential use in biomedical applications such as drug delivery and ROS therapy. For example, PEG-*b*-PDLLA stomatocytes loaded with inorganic MnO_2_ nanoparticles showed the ability to convert H_2_O_2_ into mechanical motion [83]. When tested on H_2_O_2_-exposed adenocarcinoma cells, the stomatocytes had a positive effect on the cell viability and decreased ROS induction. In another study, PEG-*b*-PS stomatocytes with platinum nanoparticles (Pt NPs) in their stomata and naphthalocyanine, a NIR light absorber, in the hydrophobic parts of the membranes were prepared [81]. They exhibited the ability to practice chemotactic-controlled movement towards H_2_O_2_ produced from human breast adenocarcinoma cells and allowed photothermal ablation and subsequent cell death when NIR light was applied. However, as for all nanocompartments, the size of the nanomotors remains an important aspect in their design for their further application in the biomedical area. Ultrasmall stomatocytes (approximately 150 nm) were prepared from PEG-*b*-PS block copolymer with encapsulated catalase in their stomata and evaluated for their ability to cross the membranes of HeLa cells [82]. It was found that they exhibited better cellular uptake than larger stomatocytes and presented the highest internalization values when incubated with the fuel, H_2_O_2_, highlighting its beneficial effect on their movement. Although the studies concerning nanomotors in biological conditions are at an early stage, they show promise for further use in biomedical applications, such as cell sorting and directional movement, taking into consideration parameters such as control over speed and direction, enzyme loading and activity, and substrate availability [175].

#### 4.2.2. In Vivo Studies

Successful in vitro evaluation of nanocompartments leads to in vivo studies for various biomedical applications and treatments, such as enzyme replacement [78,89,120,138] and gene therapy [80], cancer [76,77], hyperammonemia [95], diabetes [87], and osteoporosis [69]. When L-asparaginase was loaded in poly(ethylene glycol)-*block*-2-hydroxypropyl methacrylate (PEG-*b*-PHPMA) polymersomes, the enzyme was more protected from proteolytic attack in vitro and in vivo than native or PEGylated enzymes. In addition, the immunogenicity was significantly reduced in an immunocompetent Balb/c mouse model, making the system a promising alternative for enzyme replacement therapy [78]. For the same purpose, PMOXA-*b*-PDMS-*b*-PMOXA polymersomes, encapsulating HRP and permeabilized with an OmpF mutant, were evaluated in vitro and in vivo in zebrafish embryos, establishing their potential to act as homeostatic artificial organelles, as they preserved their structure and detoxification ability for at least 24 h (Figure 3C) [138].

Clusters of nanocompartments were evaluated for their potential in enzyme replacement therapy, as they offer the advantage of colocalization and high cell-surface binding and accumulation [120]. DNA polymersomes encapsulating laccase, an enzyme that oxidizes mainly phenolic compounds, were tested on zebrafish embryos for their stability, cluster formation, and biodistribution. Within 30 min of injection, the clusters of nanocompartments interacted with the posterior caudal vein and preserved their architecture for at least 24 h. PICsomes have also been extensively investigated for their potential in enzyme replacement and gene therapy [80,89]. PICsomes made of α,β-polyaspartic acid, PEG-*block*-poly(α,β-aspartic acid), and poly([5-aminopentyl]-α,β-aspartamide) that contained guanidinium groups were evaluated for their in vivo use [62]. It is important to mention that one of the main drawbacks to the use of PICsomes in biological conditions is their sensitivity to high ionic strength, leading to their deformation in physiological environments. However, the addition of guanidinium groups increased the stability of these PICsomes, and when injected in BALB/c mice, they performed with increased circulation time in the bloodstream and no apparent aggregation. These promising results led to their further development as a dual-delivery system with Rnase H encapsulated in their cavity and single-stranded oligonucleotides in the PICsome membrane for gene knockout therapy [80]. When tested on lung carcinoma cells, the PICsomes successfully delivered their cargo, exhibiting the desired gene knockout activity. Apart from Rnase H, two methionine γ-lyase mutants were also successfully encapsulated in PEG-Pasp- and poly-L-lysine (PLys)-based PICsomes and tested on BALB/c mice [89]. Although low percentages of enzyme encapsulation were reported (3.7% and 11%) in the blood circulation, the half-lives of the enzymes increased at least 20%, and their activity remained high for at least 24 h.

For cancer treatment, polymersomes made of poly(ethylene glycol)-*block*-thioketal-linked camptothecin methacrylate-co-2-(pentamethyleneimino) ethyl methacrylate encapsulating Gox and ultrasmall iron oxide nanoparticles (USIONs) were designed for precise cooperative cancer therapy [76]. These catalytic nanocompartments exhibited high efficacy in suppressing the tumor size in lung tumor-bearing mice, increasing the survival rate from 0% for untreated and camptothecin-treated animals to 80% in 90 days while their systemic toxicity remained low. Meanwhile, taking advantage of the overexpression of matrix metalloproteinases (MMPs) in tumor sites, polymersomes composed of triblock copolymers of MMP-cleavable peptide-linked poly(ethylene glycol), poly(ε-carprolactone) (PEG-GPLGVRG-PCL), and CPP-mimicking polymer poly(3-guanidinopropyl methacrylamide) (PGPMA) were loaded with the MMP inhibitor marimastat and colchicine, an inhibitor of microtubule formation [77]. The nanocompartments not only reduced the tumor size by almost 1.5 times in orthotopic and metastatic breast cancer-bearing mice but were successful in inhibiting its relapse after surgical abscission in almost 70% of the animals.

Polymersomes designed for the oral treatment of hyperammonemia were prepared from poly(styrene)-*block*-poly(ethylene oxide) (PS-*b*-PEO) polymersomes, encapsulating the pH-dependent fluorescent dye 8-hydroxypyrene-1,3,6-trisulfonate (HPTS), and tested on bile duct-ligated (BDL) rats [95]. These polymersomes were found to be more stable and effective for oral administration than the current liposome peritoneal treatment used in hospital settings. Meanwhile, for the treatment of diabetes mellitus, “sugar-sponges” were prepared from poly(ethylene oxide)-*block*-poly[(7-(2-methacryloyloxyethoxy)-4-methylcoumarin)-*stat*-2-(diethylamino)ethyl methacrylate-*stat*-(α-D-glucopyranosyl)ethyl methacrylate] (Figure 3D) [87]. The lectin moieties of these glycopolymersomes facilitated the uptake of glucose into their cavities when glucose concentrations were high, maintaining the blood sugar of type I diabetic KM mice at normal levels for at least 36 h. For the treatment of osteoporosis, polymersomes made from poly(ε-caprolactone)-*block*-poly[(L-glutamic acid)-*stat*-(L-glutamic acid-alendronic acid)] diblock copolymers were prepared, encapsulating β-estradiol. The presence of alendronic acid (ADA) in the outer membranes of these vesicles increased their affinity for hydroxyapatite, a mineral found in bones, by 20% and promoted osteogenic differentiation. Overall, a 70.4% recovery rate of total bone mineral density (BMD) and a 99.3% recovery rate of tibia BMD were reported after treating ovariectomized osteoporosis rats [69].

### 4.3. Vesicles as Model Systems for Organelles and Cells

While bottom-up synthetic cells have reduced complexity relative to native cells, they offer simplified views on cellular processes, supporting our understanding of complex metabolic processes. One can develop insights into the fundamental elements that control cellular behavior and function and gain greater knowledge of diseases and treatment approaches. The bottom-up strategy also offers the unique opportunity to combine artificial and biological components to create hybrid biological systems augmenting certain aspects of living systems. GUVs serve as excellent models for cells because of their size similarity and unilamellar membrane structure. There are various aspects that have to be taken into account, starting with the type of membrane and its properties up to the functionality of the whole system.

**Figure 4:**
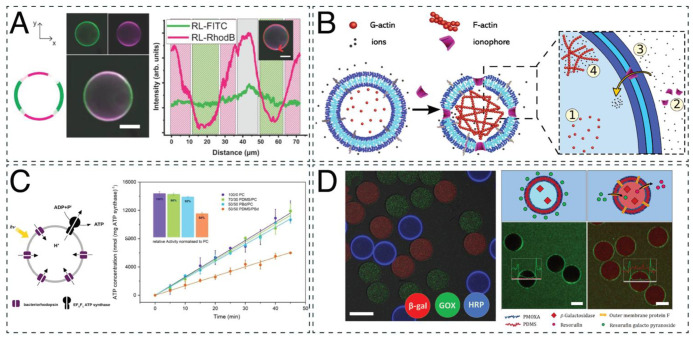
Applications of polymeric compartments to mimic cellular aspects. (**A**) Cross-sectional micrograph of DPPC-mPEG-*b*-PCL GUVs stained via fluorescein isothiocyanate (FITC, on mPEG-*b*-PCL, green) and Liss Rhod PE (in DPPC, red). Adapted with permission from [73]. Copyright 2020 MDPI. (**B**) Schematic representation of selective membrane permeabilization resulting in the formation of actin filaments triggered by ion import. Adapted with permission from [71]. Copyright 2020 John Wiley and Sons. (**C**) Schematic representation of an ATP-producing vesicle and light-driven ATP synthesis in lipid and hybrid vesicles. Adapted with permission from [154]. Copyright 2020 John Wiley and Sons. (**D**) CLSM micrograph of PMOXA-*b*-PDMS GUVs containing β-galactosidase enzymes labeled with Cy5 (red), glucose oxidase enzymes labeled with Oregon green 488 (green), and HRP enzymes labeled with Atto 550 (blue) (scale bar 50 μm). GUVs equipped with OmpF pores enabled the influx of resorufin galactopyranoside, which was converted to the fluorescent product resorufin (red) by β-galactosidase (scale bar 50 μm). Adapted with permission from [39]. Copyright 2020 John Wiley and Sons.

Lipids and lipid mixtures frequently serve as simplified models for cellular membranes [176,177,178,179,180]. However, natural cell membranes are much more complex in their composition and architecture [181]. Membrane asymmetry is an important parameter that is often overlooked but plays a major role in the function of a membrane, as it affects signal transduction and enables orientation of membrane proteins, which is crucial for their directional enzymatic activity [182,183]. Using emulsion–centrifugation, giant vesicles with an inner leaflet composed of PBd-*b*-PEO copolymer and an outer leaflet of 1-palmitoyl-2-oleoyl-sn-glycero-3-phosphocholine (POPC) were produced to mimic the asymmetry of cell membranes. However, this approach is limited to vesicles with a polymer inner leaflet and lacks long-term stability in the inverse conformation [34]. Microfluidic methods have also been employed for the high-throughput generation of asymmetric giant vesicles but have been applied only to lipid vesicles to date [184,185,186]. Cellular membranes not only show an asymmetry between leaflets but possess compositional heterogeneities and form domains in a range of sizes that can contain specific proteins important for membrane trafficking, signaling, and the assembly of specialized structures [187]. By mixing the phospholipid (PL) 1,2-dipalmitoyl-sn-glycero-3-phosphocholine (DPPC) and the semicrystalline block copolymer methoxy-poly(ethylene glycol)-*block*-poly(ε-caprolactone) (mPEG-*b*-PCL), large PL-rich and block copolymer-rich phase-separated domains and small domains of mixed PL and copolymer (Figure 4A) can be created in hybrid GUVs. The formation of crystalline PCL and gel-like DPPC regions was confirmed through X-ray scattering and diffraction. These regions strongly influenced the PL membrane fluidity and order, indirectly influencing the mechanical and permeability properties of the membrane [73]. Polymer crystallinity plays an important role in the formation of heterogeneous membranes. When mixing the noncrystalline polymer PBd-*b*-PEO with the phospholipid POPC, no evidence for the formation of lipid-rich or polymer-rich domains was found. Membrane fluidity was shown to decrease with increasing polymer fraction by a factor of five to seven, depending on the polymer length [66]. While polymeric membranes are promising candidates for the creation of artificial cell membranes, using hybrid lipid–polymer cell membrane models combines the chemical versatility and robustness of polymers with the biocompatibility of lipids and allows for the formation of membrane domains for spatial organization.

Natural membranes are constantly being remodeled in a dynamic process, enabling the adaptation of cells to their current environment [188]. Fission and fusion of vesicles are among the most important membrane remodeling processes, with the latter being promoted by the fusogenic SNARE proteins. Using SNARE proteins, membrane fusion in poly(dimethylsiloxane)-*graft*-poly(ethylene oxide) (PDMS-*g*-PEO) membranes and hybrids thereof was achieved. Through the reconstitution of SNARE proteins in polymeric membranes, a size increase and membrane protein coreconstruction using two proteins reconstructed in different polymersomes were obtained [156]. Membrane remodeling arises not only from membrane fusion but from trans-bilayer migration (flip-flop) of amphiphilic molecules [189] and was also demonstrated in asymmetric PBd-*b*-PEO/POPC membranes [34]. For those membranes, a flip-flop half-life of around 7.5 h was demonstrated, despite the size difference between the hybrid asymmetric membrane sheets, which was consistent with values reported for lipid vesicles [190,191].

Most cellular systems are compartmentalized across several length scales and sub-compartments, i.e., organelles, which are essential to spatially separate processes within cells [192]. Using multicompartmental vesicles, supramolecular assemblies with hierarchical organization, increased complexity, and subcompartments can be created. A simple example of a multicompartmentalized vesicle was obtained by (co)loading lipidic (POPC, 1,2-dimyristoyl-sn-glycero-3-phosphocholine (DMPC), phosphatidylcholine (PC), DPPC) nano-sized vesicles within PBd-*b*-PEO giant vesicles using emulsion centrifugation. Through the use of thermosensitive lipids with different release temperatures, a simple microreactor with the ability to externally stimulate contained cascade reactions on demand was created [34]. In another example, multicompartmental GUVs were formed by the encapsulation of responsive nanoparticles loaded with biomolecules inside GUVs. The nanoparticles were loaded with either enzyme substrates or biopores and disassembled in the presence of dithiothreitol (DTT), thus releasing the nanoparticles’ cargo. The release of the enzyme substrates catalyzed the reaction of coencapsulated enzymes, while the release of the ion channels (gramicidin) allowed them to integrate into the vesicle membrane, causing controlled permeabilization (Figure 4B). Using this approach, a multicompartment cellular system was created that was able to change membrane permeability upon external signals, a process naturally occurring in neurons [92]. An evolution of that study examined these multicompartmental vesicles encapsulated with two different subcompartments: reduction-sensitive nanoparticles and non-reduction-sensitive heparin micelles. Upon reductive external stimuli, signaling cascades were triggered that led to the assembly of a cytoskeleton in the form of an actin network within GUVs while maintaining internal compartmentalization through reduction-insensitive compartments [71].

To understand the interaction mechanisms of cells in tissues from a bottom-up perspective, artificial cells can be assembled into tissues. Simple artificial tissues comprising GUV aggregates segregated by an artificial membrane can be used for this purpose. Using a DNA-based linking system, a multicompartment GUV network containing a mixture of GUVs, each composed of either Pluronic L121, PBd-*b*-PEG, or polylactic acid-*block*-polyethylene glycol (PLA-*b*-PEG), served for the selective assembly of polymeric vesicles. The functionalization of GUVs with cholesterol-tagged DNA mediated the linkage of the vesicles and resulted in deformation at the adhesion site. The thermal regulation of the DNA hybridization allowed the linkage that controlled the contact area between the GUVs to be switched on and off [37].

Assembling polymeric and lipidic vesicles into hierarchical structures is a promising strategy to establish cell or organelle models closer to nature. However, controlling the encapsulation of smaller vesicular structures is challenging, as it is often governed by stochastic processes, and control over the encapsulation efficiency is limited. Precise control over the encapsulated cargo and the influence thereof is essential for the creation of model systems. While membranes serve a very important role in separating aqueous compartments from their surroundings, selective transport of certain molecules across a bilayer membrane is a key requirement for any cell. A straightforward method for introducing selective transport to hybrid GUVs is through the use of a permeable polymer. The block copolymer oligo(aspartic acid)-*block*-poly(propylene oxide) forms polymer-rich domains in a polymer/lipid hybrid membrane that are intrinsically permeable to small molecules, but the selectivity and specificity of the transport across the membrane is limited. A more challenging approach is the insertion of pores or membrane proteins into synthetic bilayer membranes to induce selective permeability. Through film rehydration, membrane proteins can be directly added to the rehydration buffer, as shown for the model biopore OmpF [90] or the membrane proteins ATP synthase and cytochrome *bo*_3_ ubiquinol oxidase [156]. The addition of membrane proteins to preformed vesicles presents an alternative means of protein incorporation and has been shown for OmpF and gramicidin in microfluidic double emulsion templated PMOXA-*b*-PDMS GUVs [39,68].

In cells, a majority of the energy used for chemical work is stored in the form of adenosine triphosphate (ATP). ATP drives a plethora of energy-consuming processes within a cell, such as protein biosynthesis, motility, membrane transport, and intracellular signaling. In eukaryotic cells, most ATP is produced in the mitochondrion [193], making it a biologically relevant organelle to mimic. One popular approach for this is the reconstruction of a proton pump within a polymeric or hybrid polymer/lipid membrane to generate a proton gradient that can subsequently power a reconstructed (F_0_F_1_-)ATP synthase for the production of ATP from adenosine diphosphate (ADP) and inorganic phosphate (P_i_). This has been shown using polymersomes and GUVs made from the graft polymer PDMS-*g*-PEO and the diblock copolymer PBd-*g*-PEO (Figure 4C) [72,154]. PDMS-*g*-PEO and PDMS-*g*-PEO/PC hybrid polymersomes and GUVs served as the base for directional insertion of a proton pump into the membrane and subsequent lowering of pH inside the GUV through active transport of protons across the polymer bilayer. Rheological testing revealed that PDMS-*g*-PEO and hybrid membranes showed higher flexibility than pure PC membranes and that the insertion of protein pores into the membrane further decreased rigidity and increased membrane fluidity. The high fluidity of the copolymer was attributed to the fact that the molecular weight of the PDMS was inversely proportional to the membrane’s diffusion coefficient [194]. In a similar study, the transmembrane protein F_0_F_1_-ATP synthase and the light-sensitive proton pump bacteriorhodopsin were integrated into the membranes of PDMS-*g*-PEO, PBd-*b*-PEO, and polymer/lipid hybrid polymersomes. This study demonstrated enhanced long-term stability of the membrane proteins as compared with liposomes, with the highest activity and longest stability in PDMS-containing membranes [154].

Using microfluidic double emulsion templated GUV formation, bioactive cargo has been encapsulated with an encapsulation efficiency of up to 99%. Through pore formation using OmpF, catalytic GUVs were produced to create a three-step enzymatic cascade that converted fluorescein di-β-galactopyranoside (FGD) into resorufin via two intermediate steps, demonstrating a simple intracellular communication pathway (Figure 4D) that could be extended in a modular fashion [39]. Through the encapsulation of bioactive molecules, e.g., enzymes, simple model artificial cells with in situ enzymatic activity have been created that could convert substrates from outside of the GUVs. While the complete removal of organic solvent is not always trivial, this method allows for the efficient, high-throughput production of vesicles with precise control over lumen and membrane composition, which is essential for the applicability of GUVs as cell mimics [195].

## 5. Conclusion and Perspectives

Polymeric micro- and nanocompartments offer great versatility; they can be loaded with various functional molecules, their surfaces can be functionalized using targeting moieties, and their membranes can gain specific functions using membrane proteins. Polymer vesicles have been studied extensively, as they offer several advantages over liposomes, such as increased chemical versatility and mechanical stability, and have found applications in imaging, therapeutics, and creation of artificial cellular models.

Nanoscale polymeric vesicles, including clusters of nanocompartments, have been applied for imaging and theranostics by combining therapeutic and imaging properties. By encapsulating imaging probes (e.g., quantum dots, fluorescent dyes) and/or therapeutic compounds, potentially toxic or quenching side effects can be avoided while maintaining observable, targeted delivery. Similar approaches have been applied for therapeutic applications; cargo can be encapsulated within their cavities or loaded within their structure to increase stability, activity, and blood circulation. Specifically, through the permeabilization of enzyme-loaded polymersomes using pore proteins or pore-forming peptides, catalytic nanocompartments can be created that perform catalytic activity. This can apply to enzyme replacement therapy or on-demand drug production.

Polymeric microvesicles have mainly been used for cellular modeling and the creation of artificial cells. Using polymeric or hybrid polymer/lipid membranes, natural membranes can be mimicked in terms of membrane asymmetry or domain formation. Through reconstitution of membrane proteins, cellular signaling can be studied, and by combining nano- and micrometer-sized vesicles, structures with complex hierarchies can be built that mimic natural compartmentalized systems. Using both lipids and polymers allows for the exploitation of the advantages of both types of amphiphiles, facilitating membrane protein reconstitution while increasing the compartment’s stability. Attempts have been made to mimic cytoskeleton networks, cellular movement, and inter- and intracellular communication. However, progress still needs to be made in order to gain understanding and to realize the full potential of artificial cell models.

Progress in polymer chemistry has enabled the creation of polymers with tailored properties, and because of the immense variety of chemical compositions and functionalizations, polymers can be tailored to improve biocompatibility, biodegradability, and toxicity. Even so, the production of compartments thereof is still challenging; most of the fabrication techniques are laborious and low throughput, which also complicates subsequent scale-up attempts. Using microfluidic methods enables the high-throughput production of polymeric vesicles; however, examples are still limited, in the early stages of development, and mainly applied to micrometer-sized compartments. Even though significant progress has been made in the formulation of catalytic compartments and control over the encapsulation efficiency, surface functionalization, and membrane protein reconstitution, further research is still needed to obtain insight into the structure and behavior of said compartments in a biomedical context. Hybrid materials are being investigated with significant progress; however, research is still needed to study interactions of polymeric and biological materials in order to increase biocompatibility. For therapeutic applications, most of the studies presented tested in vitro, and the in vivo studies were limited to mice. While most studies presented showed promising results, tests for the efficacy and compatibility of polymer compartments are necessary for their effective application in a medical context. Employing multifunctional materials is expected to increase applicability of polymeric compartments for patient-oriented medical strategies; incorporation of multistimulus-responsive materials that activate their functionality in response to multiple intracellular or external signals would increase the number of applications of polymeric compartments in the biomedical field.

## Figures and Tables

**Table 2:** Biomolecules and their applications in compartments.

Biomolecule	Polymer	Location in Assembly	Application/Function
Actin	PMOXA-*b*-PDMS-*b*-PMOXA [71]	Encapsulated (in GUVs)	Polymerization to form a cytoskeleton
ATP synthase	PDMS-*g*-PEO, PBd-*b*-PEO [72,154]	Incorporated within membrane (GUVs)	ATP generation
Bacteriorhodopsin	PDMS-*g*-PEO, PBd- *b*-PEO [154]	Incorporated within membrane (GUVs)	Pumping protons across membrane
Catalase	PEG-*b*-PS [82], PAH and DEX [59]	Encapsulated with the stomata of polymer stomatocytes and LbL capsules	Conversion of hydrogen peroxide to oxygen and water for self-propelled movement
Cholesterol–DNA	PEO-*b*-PPO-*b*-PEO (Pluronics L121), PBd-*b*-PEG, PLA-*b*-PEG [37]	Incorporated within membrane (GUVs)	Clustering of polymersomes
Cytochrome *bo_3_* ubiquinol oxidase (Cyt *bo*_3_)	PBd–PEO:POPC hybrid [155], PDMS-*g*-PEO and PDMS-*g*-PEO/PC hybrid [72,156]	Incorporated within membrane (polymersomes, GUVs)	Pumping protons across membrane
DNA nanopore NP-3c	PMPC-*b*-PDPA [93]	Incorporated within membrane (GUVs)	Pore formation for cross-membrane diffusion
Dopa decarboxylase (DDC)	PMOXA-*b*-PDMS [157]	Encapsulated (in polymersomes)	Production of dopamine
Erythrosine B (and its ester derivatives)	F127 Pluronic (mixed with DPPC lipids) [158]	Incorporated within membrane (polymersomes)	Photodynamic therapy
Glucose oxidase (Gox)	PMOXA-*b*-PDMS [39,91], PEG-*b*-P(CPTKMA-*co*-PEMA) [76], PATK and PEG-*b*-Pasp [65]	Encapsulated (in GUVs, polymersomes, and PICsomes)	Catalysis of glucose oxidation to hydrogen peroxide and D-glucono-δ-lactone
Gramicidin	PMOXA-*b*-PDMS [39], PMOXA-*b*-PDMS-*b*-PMOXA [71]	Incorporated within membrane	Membrane permeabilization towards ions
Horseradish peroxidase (HRP)	PMOXA-*b*-PDMS [39], PMOXA-*b*-PDMS-*b*-PMOXA [90,138], carbohydrate-*b*-PPG [54]	Encapsulated (in GUVs, polymersomes, capsosomes)	Catalysis of oxidation of organic substrates by hydrogen peroxide
Icosane	PAA and PAH (LbL) [58]	Encapsulated (in capsules)	Acting as a phase change material for thermal energy storage
Inducible nitric oxide synthase (iNOS)	PMOXA-*b*-PDMS-*b*-PMOXA [159]	Encapsulated (in polymersomes)	Oxidation of l-arginine to l-citrulline and nitric oxide (NO)
Ionomycin	PMOXA-*b*-PDMS-*b*-PMOXA [71]	Incorporated within membrane	Membrane permeabilization towards ions
Laccase	PMOXA-*b*-PDMS [120]	Encapsulated (in polymersomes)	Oxidation of phenolic and nonphenolic compounds
Lactoperoxidase (LPO)	PMOXA-*b*-PDMS [91]	Encapsulated (in polymersomes)	Oxidation of Amplex red using hydrogen peroxide
L-asparaginase	PMPC-*b*-PDPA and PEO-*b*-PBO [84], PEG-*b*-Pasp and P(Asp-AP) [64], PEG-*b*-PHPMA [78]	Encapsulated (in polymersomes, PICsomes)	Catalysis of L-asparagine to l-aspartic acid and ammonia
Lipase	PMOXA-*b*-PDMS-*b*-PMOXA [71]	Encapsulated (in polymersomes)	Catalysis of the hydrolysis of fats
Luciferase	PMOXA-*b*-PDMS [160]	Encapsulated (in polymersomes)	Bioluminescence
Melittin	PMOXA-*b*-PDMS [91,144,161]	Incorporated within membrane (polymersomes, GUVs)	Pore formation for cross-membrane diffusion
Methionine γ-lyase (MGL)	PEG-P(Asp) and PLys [63,89]	Encapsulated (in PICsomes)	Cancer therapy
Outer membrane protein F from *E. coli* (OmpF)	PMOXA-*b*-PDMS [39], PMOXA-*b*-PDMS-*b*-PMOXA [90,138]	Incorporated within membrane (GUVs, polymersomes)	Pore formation for cross-membrane diffusion
Penicillin acylase	PMOXA-*b*-PDMS-*b*-PMOXA [162]	Encapsulated (in polymersomes)	Production of antibiotic cephalexin
Rnase H	PEG-*b*-polypeptide (with single-stranded oligonucleotides) [80]	Encapsulated (in PICsomes)	Gene knockout therapy
β-galactosidase	PMOXA-*b*-PDMS [39] carbohydrate-*b*-PPG [54]	Encapsulated (in GUVs, capsosomes)	Catalysis of the hydrolysis of β-galactosides into monosaccharides
β-glucuronidase	PMOXA-*b*-PDMS [161]	Encapsulated (in polymersomes)	Cleavage of the glucuronide moiety from glucuronide-conjugates
Soluble guanylyl cyclase (sGC)	PMOXA-*b*-PDMS-*b*-PMOXA [159]	Encapsulated (in polymersomes)	Production of cyclic 3,5-guanosine monophosphate (cGMP)
Trypsin	PMPC-*b*-PDPA [93]	Encapsulated (in polymersomes)	Hydrolyzation of proteins
Tyrosinase	PMOXA-*b*-PDMS [163]	Encapsulated (in polymersomes)	Oxidation of L-DOPA
Urate oxidase (UOX)	PMOXA-*b*-PDMS-*b*-PMOXA [16]	Encapsulated (in polymersomes)	Production of hydrogen peroxide for a cascade reaction

## Data Availability

Not applicable.

## Abbreviations

ADA	alendronic acid
ADP	adenosine diphosphate
AP	poly([5-aminopentyl]-α,β-aspartamide)
ATP	adenosine triphosphate
BDL	bile duct-ligated
BMD	bone mineral density
cGMP	cyclic guanosine monophosphate
CLSM	confocal laser scanning microscope
CMA	7-(2-methacryloyloxyethoxy)-4-methylcoumarin
CNC	catalytic nanocompartment
CPP	cell-penetrating peptide
CPT	camptothecin
CPTKMA	thioketal-linked CPT methacrylate monomer
cRGD	cyclic arginine-glycine-aspartic
CT	computed tomography
CTAB	cetyltrimethylammonium bromide
DBCO	dibenzocyclooctyne
DDC	dopa decarboxylase
DEA	2-(diethylamino)ethyl methacrylate
DEX	dextran sulfate
DMPC	1,2-dimyristoyl-sn-glycero-3-phosphocholine
DNA	deoxyribonucleic acid
DPPC	1,2-dipalmitoyl-sn-glycero-3-phosphocholine
DTT	dithiothreitol
*E. coli*	*Escherichia coli*
FGD	fluorescein di-β-galactopyranoside
FITC	fluorescein isothiocyanate
FRAP	fluorescence recovery after photobleaching
GEMA	(α-d-glucopyranosyl)ethyl methacrylate
Gox	glucose oxidase
GUV	giant unilamellar vesicle
HPTS	8-hydroxypyrene-1,3,6-trisulfonate
HRP	horseradish peroxidase
iNOS	nitric oxide synthase
ITO	indium tin oxide
LbL	layer-by-layer
L-DOPA	levodopa/l-3,4-dihydroxyphenylalanine
LPO	lactoperoxidase
MMP	matrix metalloproteinase
mNSS	modified neurological severity scores
mPEG	methoxy-poly(ethylene glycol)
MRI	magnetic Resonance Imaging
NgR	Nogo-66 receptor
NIR	near-infrared
NP	nanoparticle
OEGMA	oligo(ethylene glycol) methyl ether methacrylate
OmpF	outer membrane protein F (from *Escherichia coli*)
PA	poly-L-arginine
PAA	poly(acrylic acid)
PAH	poly(allylamine hydrochloride)
PAMAM	poly(amidoamine)
PAsp	poly(α,β-aspartic acid)
PATK	poly([2-[[1-[(2-aminoethyl)thio]-1-methylethyl]thio]ethyl]-α,β-aspartamide)
PBd	poly(1,2-butadiene)
PBO	poly(butylene oxide)
PBzMA	poly(benzyl methacrylate)
PC	phosphatidylcholine
PCL	poly(ε-caprolactone)
PDA	polydopamine
PDLLA	poly(D,L-Lactic Acid)
PDMS	poly(dimethylsiloxane)
PDPA	poly [2-(diisopropylamino)ethyl methacrylate]
PEG	poly(ethylene glycol)
PEHO	poly(3-ethyl-3-hydroxymethyloxetane)
PEI	poly(ether imide)
PEMA	poly(ethyl methacrylate)
PEO	poly(ethylene oxide)
PEtOz	poly(2-ethyl-2-oxazoline)
PG	poly(glycidol)
PGPMA	poly(3-guanidinopropyl methacrylamide)
PHPMA	poly(*N*-(2-Hydroxypropyl) methacrylamide)
P_i_	inorganic phosphate
PiB	polyisobutylene
PIC	polyion complex
PISA	polymerization-induced self-assembly
PL	phospholipids
PLA	polycaprolactone
PLys	poly-lysine
PMA	polymethyl acrylate
PMOXA	poly(2-methyl-2-oxazoline)
PMPC	poly(2-methacryloyloxyethyl phosphorylcholine)
POEGMA	poly(oligo(ethylene glycol) methyl ether methacrylate)
POPC	1-palmitoyl-2-oleoyl-sn-glycero-3-phosphocholine
PS	polystyrene
PSS	poly(styrene sulfonate)
PSMA	poly(stearyl methacrylate)
PPG	poly(propylene glycol)
PPO	poly(p-phenylene oxide)
PTMC	tetraphenylethylene pyridinium modified trimethylenecarbonate
PVP	polyvinylpyrrolidone
P(CLgTMC)	poly(caprolactone-gradient-trimethylene carbonate)
ROS	reactive oxygen species
*S. aureus*	*Staphylococcus aureus*
sGC	soluble guanylyl cyclase
siRNA	small interfering ribonucleic acid
SPAAC	strain-promoted azide-alkyne cycloaddition
SPECT	single-photon emission computed tomography
SPIONs	superparamagnetic iron oxide nanoparticles
ssDNA	single-stranded deoxyribonucleic acid
TA	tannic acid
TTA-UC	triplet–triplet annihilation based molecular photon upconversion
UOX	urate oxidase
USIONs	ultrasmall iron oxide nanoparticles
VBA	poly(vinyl benzaldehyde)
